# COVID-19 cutaneous manifestations in children and adolescents: a systematic review

**DOI:** 10.1590/1984-0462/2022/40/2021134IN

**Published:** 2022-06-10

**Authors:** Ricardo Pasquini, Felipe Antonio Torres Mazzo, Fernanda de Almeida Vieira, Gustavo de Souza Bueno, João Vitor Correa Previdi, Lara Rozetti da Silva, Nasthia Kreuz Baziulis da Silva, Joseph Lucius Jorizzo, Felipe Bochnia Cerci

**Affiliations:** a Pontifícia Universidade Católica do Paraná, Curitiba, PR, Brazil.; b Wake Forest University, Winston-Salem, NC, United States of America.; c Weill Cornell Medical College, New York, NY, United States of America.; d Universidade Federal do Paraná, Curitiba, PR, Brazil.; e Clínica Cepelle, Curitiba, PR, Brazil.

**Keywords:** COVID-19, SARS-CoV-2, Skin manifestations, Children, Adolescents, Pediatrics, COVID-19, SARS-CoV-2, Manifestações cutâneas, Criança, Adolescente, Pediatria

## Abstract

**Objective:**

The aim of this study was to evaluate the coronavirus disease 2019 (COVID-19) cutaneous manifestations described in pediatric patients and discuss their relevance for early diagnosis.

**Data source:**

The study consisted of a systematic review of original articles indexed in PubMed and Embase databases, as well as gray literature articles found through Google Scholar. A search strategy, based on PICO (Patient, Intervention, Comparison, Outcome) Tool, with the terms “child,” “infant,” “childhood,” “adolescents,” “teenagers,” “COVID-19,” “SARS-CoV-2,” and “skin manifestations,” was performed to optimize the findings. The study did not restrict any article regarding language.

**Data synthesis:**

Out of the 310 articles that initially met the inclusion criteria, 35 were selected for review, totalizing 369 patients. The most common COVID-19 cutaneous manifestations in children and adolescents were Chilblain-like lesions, presented in 67.5% of the cases, followed by erythema multiforme-like (31.7%) and varicella-like lesions (0.8%). The Chilblain-like lesions appeared 7.6 days (95%CI 7.4–7.8) after the viral infection and lasted for 17.5 days (95%CI 16.5–18.5), erythema multiforme-like lesions appeared in 9.5 days (95%CI 9–10) and lasted for 10.3 days (95%CI 9.1–11.5), and varicella-like lesions appeared in 12.3 days (95%CI 4–20.6) and lasted for 7 days.

**Conclusions:**

Knowledge of the different skin manifestations in children and adolescents with COVID-19 is essential for an early diagnosis and, consequently, the possibility of promptly care adoption as well as to interrupt the new coronavirus transmission chains in the current pandemic context.

## RESUMO

**Objetivo:**

Avaliar as manifestações cutâneas da Doença de Coronavírus 2019 (COVID-19) descritas em pacientes pediátricos e discutir sua relevância para o diagnóstico precoce.

**Fontes de dados:**

O estudo consistiu em uma revisão sistemática de artigos originais indexados nas bases de dados PubMed e Embase, assim como na literatura cinzenta localizados via Google Scholar. Uma estratégia de busca, baseada na *PICO (Patient, Intervention, Comparison, Outcome) Tool,* com os termos “child”, “infant”, “childhood”, “adolescents”, “teenagers”, “COVID-19”, “SARS-CoV-2” e “skin manifestations” foi conduzida para otimizar os achados. Não houve restrições de idiomas.

**Síntese de dados:**

35 dos 310 artigos que inicialmente cumpriram os critérios de inclusão foram selecionados para revisão, totalizando 369 pacientes. As manifestações cutâneas mais comuns de COVID-19 em crianças e adolescentes foram as lesões *Chilblain-Like* presentes em 67,5% dos casos, seguidas pelas lesões *Eritema Multiforme-Like* (31,7%) e *Varicella-Like* (0,8%). As lesões *Chilblain-Like* surgiram 7,6 dias (IC95% 7,4–7,8) após a infecção viral e tiveram duração de 17,5 dias (IC95% 16,5–18,5); as lesões *Eritema Multiforme-Like* surgiram em 9,5 dias (IC95% 9–10) e duraram 10,3 dias (IC95% 9,1–11,5) e as lesões *Varicella-Like* apareceram em 12,3 dias (IC95% 4–20,6) e duraram 7 dias.

**Conclusões:**

O conhecimento das diferentes manifestações cutâneas em crianças e adolescentes com COVID-19 é essencial para o diagnóstico precoce da doença, assim como possibilitar adoção de prontos cuidados e interromper cadeias de transmissão do novo coronavírus.

## INTRODUCTION

Pediatric patients correspond to 1–5% of severe acute respiratory syndrome coronavirus 2 (SARS-CoV-2) confirmed cases.^1,2^ Dermatological manifestations in pediatric patients appear during the most critical transmissible stages of the disease.^3^ Identifying these lesions is essential for early diagnosis of coronavirus disease 2019 (COVID-19) because children and adolescents have milder clinical features than adults.^3–6^ Similarly to the adults, the pediatric population may also have exacerbation of inflammatory cytokines, a potential cause of severe hyperinflammatory syndromes.^6,7^

Cutaneous manifestations have been increasingly reported in association with COVID-19. Although there is significant relevance of the theme for pediatric health care, much remains unknown regarding the characterization and incidence of these dermatological symptoms in children and adolescents. The present systematic review aims to evaluate the COVID-19 cutaneous manifestations described in pediatric patients and discuss their relevance for early diagnosis. This study addresses exclusively the pediatric population providing scientific evidence for the development of new COVID-19 guidelines.

## METHOD

This study is a systematic review of original indexed articles from PubMed and Embase databases, as well as gray literature articles found through Google Scholar.

The authors conducted a search strategy based on the PICO (Patient, Intervention, Comparison, Outcome) Tool to optimize the identification of articles in the databases and gray literature. The terms “child,” “infant,” “childhood,” “adolescents,” and “teenagers” were used to describe the study population; “COVID-19” and “SARS-CoV-2” were used to describe the study's intervention; and “skin manifestations” was used as the study's outcome. All terms mentioned were used as MeSH terms, Title/Abstract, and Text-Wide Free Terms.

Boolean operators combined theses terms in the search strategy. The “OR” operator was used between terms within the same PICO category, and the “AND” operator among terms of different PICO categories. The complete search strategy performed in this systematic review is available with the corresponding author.

The search strategy found 285 studies in the databases (77 in PubMed and 208 in Embase) and 25 articles in the gray literature. The authors removed 118 duplicates, and the remaining articles (192) were selected based on the inclusion and exclusion criteria.

This study included articles that described cutaneous manifestations in the pediatric population. Articles that did not confirm the diagnosis of COVID-19 with reverse transcription polymerase chain reaction (RT-PCR) and serology were excluded. The study did not restrict any article regarding language.

**Figure 1:**
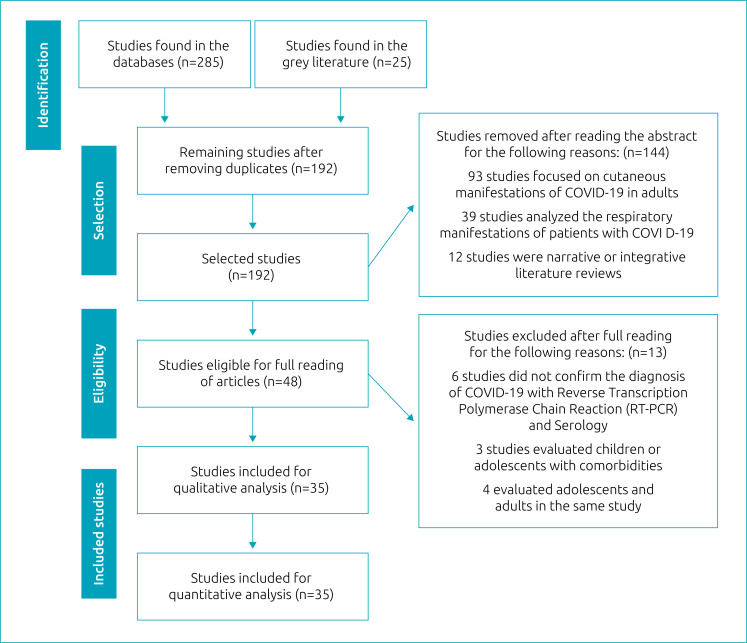
Flow diagram of the article selection process in the literature (PRISMA Flowchart).

Following the PRISMA 2020 Flowchart, the authors read the abstracts and elected 48 articles for full reading of the articles. At this stage, 144 articles were excluded. The main reasons for exclusion were that 93 articles exclusively focused on manifestations of COVID-19 in adults, not including children or adolescents; 39 articles analyzed the respiratory manifestations of the disease; 12 articles were narrative or integrative literature reviews. Then, a complete reading of the previously selected articles was carried out. At this stage, 13 studies were excluded — six of them did not confirm the diagnosis of COVID-19 with RT-PCR and serology, three evaluated children and adolescents with comorbidities and four evaluated adolescents and adults in the same study, not being possible to interpret them exclusively. At the end of this step, 35 articles were included for qualitative and quantitative analysis of this systematic review (Figure 1).

Based on PRISMA 2020 protocol, two authors independently selected the articles during each stage of the pairs’ selection process. A discussion with a third author solved disagreements between these authors. Cohen's kappa coefficient was 0.9. The National Institutes of Health (NIH) Study Quality Assessment Tool was used to assess the quality of selected studies.

Data extraction was also carried out by independent authors, in pairs, and a third author solved disagreements. Authors tabled the articles extracted data with the following variables: “cutaneous manifestation,” “country,” “number of patients and gender,” “mean age of patients,” “lesion location,” “mean time to lesion's manifestation,” “lesion mean duration,” and “histological findings on biopsy.”

Initially, all variables mentioned were analyzed descriptively. The quantitative variables were analyzed by means, medians, and standard deviations, and qualitative variables were analyzed by absolute and relative frequencies. The level of significance accepted for the statistical tests was 5%. *Statistical Package for the Social Sciences* version 27.0 software carried out the statistical analysis.

## RESULTS

Thirty-five articles, totalizing 369 patients (221 males and 148 females), were included. The demographic distribution was represented by 173 patients from Italy, 163 from Spain, 16 from France, 9 from the United States, 2 from the United Kingdom, 2 from Iran, 1 from Morocco, 1 from Russia, 1 from Israel, and 1 from Turkey. Of the selected studies, 19 were case reports, 8 were case series, 4 were cohorts, 3 were cross-sectional, and 1 was case-control.

This systematic review analyzed variables corresponding to the “lesion location,” “mean time to lesion's manifestation,” “lesion mean duration,” “mean age of patients,” and “patients’ gender” of each COVID-19 pediatrics cutaneous manifestations. The variables “mean age of patients,” “mean time to lesion's manifestation,” and “lesion mean duration” were calculated from weighted average considering the number of patients in each study as the weight for calculation. Statistical analysis disregarded articles that did not mention these events.

Chilblain-like lesions were described in 18 (51.4%) articles, totalizing 249 (67.5%) patients. All studies that considered Chilblain-like lesions described extremities involvement. As for the time of appearance and duration of the dermatological symptoms, eight and six articles addressed these topics, respectively (Table 1).

**Table 1:** Publications on COVID-19 cutaneous manifestations in pediatrics.

Cutaneous manifestation	References	Country	Number of patients and gender	Mean age of patients (years)	Lesion location	Mean time to lesion's manifestation (days)	Lesion mean duration (days)
Chilblain-like	8–14	Italy	33 Females 55 Males	11.7	88 Extremities	13.9	8.1
	15–22	Spain	58 Females 95 Males	13.4	153 Extremities	7	18
	23	The United States	1 Female 5 Males	15	6 Extremities	7	Not mentioned
	24	Israel	1 Male	16	1 Extremity	Not mentioned	Not mentioned
	25	Morocco	1 Male	17	1 Extremity	4	Not mentioned
Erythema multiforme-like	26–28	Italy	38 Females 45 Males	14	63 Extremities 1 Generalized	9.9	Not mentioned
	22, 29–31	Spain	5 Females 5 Males	9.6	2 Extremities 8 Generalized	9	10.4
	32, 33	The United States	2 Males 1 Female	2.3	3 Extremities	9	Not mentioned
	34	France	8 Females 8 Males	10	Not mentioned	Not mentioned	Not mentioned
	35	Turkey	1 Female	3	1 Generalized	5	Not mentioned
	36, 37	United Kingdom	2 Males	12.5	2 Generalized	21.5	13
	38	Iran	1 Male	1	1 Generalized	2	11
	39	Russia	1 Female	12	1 Generalized	3	3
Varicella-like	40, 41	Italy	1 Female 1 Male	8	2 Generalized	4	7
	42	Iran	1 Female	9	1 Generalized	30	7

Erythema multiforme-like lesions were mentioned in 15 (42.9%) articles, totalizing 117 (31.7%) patients. Of the studies that considered erythema multiforme-like lesions, 13 identified their location; 9 pointed to generalized, while 4 pointed to extremities involvement. As for the time of appearance and duration of the skin manifestations, 11 and 8 articles addressed these topics, respectively (Table 1).

Garcia-Lara et al. reported in the same study the presence of 25 cases of Chilblain-like lesions and 2 cases of erythema multiforme-like lesions.^22^ Thus, for tables organization, the authors placed Chilblain- and erythema multiforme-Like lesions information in different lines, each one corresponding to the specific cutaneous manifestation characteristics.

Varicella-like lesions were described in 3 (8.6%) studies, totalizing 3 (0.8%) patients. All studies that considered varicella-like lesions described that they had generalized involvement. As for the time of appearance and duration of the cutaneous manifestations, the three articles addressed both topics (Table 1).

Biopsies were carried out by 11 articles — 9 regarding Chilblain-like lesions and 2 erythema multiforme-like lesions. No studies performed biopsies in varicella-like lesions. The histological findings on COVID-19 cutaneous manifestations biopsies in pediatrics are described in Table 2.

**Table 2:** Histological findings on COVID-19 cutaneous manifestations biopsies in pediatrics.

Cutaneous manifestation	References	Histological findings on biopsy
Chilblain-like	9	Papillary dermis edema, perivascular and perieccrine lymphocytic infiltrate.
	14	Dense perivascular and periadnexal lymphocytic infiltrates that extend to the subcutaneous region.
	17	Partial epidermal necrosis and perivascular lymphocytic infiltrate in the dermis. Capillaries in papillary dermis with microthrombi and erythrocyte extravasation.
	18	Moderate edema in the papillary dermis, perivascular infiltrate, lymphocytic vasculitis infiltrate. Perieccrine and perivascular infiltrate.
	19	Vacuolar changes in the basal layer, spongiosis, dermal edema, perivascular inflammation.
	20	Basal layer vacuolar degeneration with sparse necrotic keratinocytes and perivascular lymphocytic infiltrate, without thrombosis evidence.
	21	Perivascular and periadnexal lymphocyte infiltrate, dermal papillary edema, vacuolar degeneration of the basal layer, and lymphocytic exocytosis for the epidermis and across syringeal.
	23	Perivascular lymphocytic infiltrate and superficial and lymphocytic vasculitis, without thrombosis evidence.
	24	Extensive epidermal and dermal necrosis with thrombi in vessels. Neutrophilic infiltration and nuclear debris surrounding the vessels.
	8, 10–13, 15, 16, 22, 25	Not performed
Erythema multiforme-like	27	Papillary dermis edema and erythrocytes extravasation. Perivascular lymphocytic infiltrate.
	30	Spongiosis and lymphocyte exocytosis. Perivascular lymphocytic infiltrate without eosinophils or necrotic keratinocytes. Vascular ectasia and lymphocytic vasculitis. Fibrinoid necrosis and thrombosis were absent.
	22, 26, 28, 29, 31–39	Not performed
Varicella-like	40–42	Not performed

The National Institutes of Health (NIH) Study Quality Assessment Tool^43^ demonstrated 22 (63%) studies with a good-quality rating, 10 (29%) studies with a fair rating, and 3 (8%) studies with a poor-quality rating. The rating on these studies is attributed primarily to inadequate descriptions of cases. Two authors independently assessed the quality of selected articles. A discussion with a third author solved disagreements between these authors. Table 3 illustrates the quality rating of each study design included in the systematic review based on the NIH Study Quality Assessment Tool.^43^

**Table 3:** The NIH Study Quality Assessment Tool of each study design included in the systematic review.

Study design	References	Quality rating
Case reports	8, 9, 14, 25, 29, 31, 33, 36–40, 42	Good
	17, 24, 26, 32, 35	Fair
	15	Poor
Case series	19, 23, 30, 41	Good
	16, 18, 20	Fair
	10	Poor
Cohorts	21, 22, 28	Good
	34	Poor
Cross-sectional	11, 12	Good
	13	Fair
Case-control	27	Fair

Chilblain-like lesions were the most common cutaneous manifestation in children and adolescents with COVID-19 (67.5% of patients). The erythema multiforme-like lesions affected 31.7% of patients, and the varicella-like lesions to 0.8% of cases. The major particularities of each COVID-19 cutaneous manifestations are summarized in Table 4. The prevalence described by this review in Table 4 was estimated among pediatric patients who had cutaneous manifestations and not in the pediatric population who had COVID-19.

**Table 4:** Major particularities of COVID-19 cutaneous manifestations in pediatrics.

Cutaneous manifestation of COVID-19	Percentage of cases reported	Lesion location	Mean time to lesion's manifestation (days)	Lesion mean duration (days)	Mean age of patients (years)	Patients’ gender
Chilblain-like	67.5%	100% Extremities	7.6 (95%CI 7.4–7.8)	17.5 (95%CI 16.5–18.5)	13 (95%CI 12.9–13.1)	37% Female 63% Male
Erythema multiforme-like	31.7%	83% Extremities 17% Generalized	9.5 (95%CI 9–10)	10.3 (95%CI 9.1–11.5)	12.5 (95%CI 12.3–12.7)	44% Female 56% Male
Varicella-like	0.8%	100% Generalized	12.3 (95%CI 4–20.6)	7	8.5 (95%CI 8–9)	67% Female 33% Male

95%CI: 95% confidence interval.

## DISCUSSION

Dermatological manifestations can be part of COVID-19 clinical spectrum.^44^ Their presence in pediatric patients may indicate active disease associated with high transmissibility of the virus.^45^ The incidence of these skin lesions in the pediatric population is not well characterized.^46^ Although some studies pointed to a higher incidence of urticarial lesions and maculopapular rash in children infected by the new coronavirus,^47^ this review identified that the Chilblain-like lesions are the most common cutaneous manifestation associated with COVID-19 considering children and adolescents.

Chilblain-like lesions, also known as COVID toes, are a well-described dermatosis characterized by erythema and swelling localized to acral areas, occurring most commonly on the toes and fingers.^8–25^ The second most common cutaneous manifestation related to COVID-19 was erythema multiforme-like lesions. Erythema multiforme is an acute, self-limited, manifestation that is considered to be a type IV hypersensitivity reaction. Lesions often start on the extremities and evolve into pathognomonic target or iris lesions, within a 72-h period.^22,26–39^

Another manifestation presented in the literature was the varicella-like lesions described as a generalized papulovesicular skin eruption. These lesions were initially erythematous papules, with a tendency to superficial vesiculation leading to crust formation.^40–42^

The SARS-CoV-2 virus accesses the epithelial tissue based on the mechanism of binding the viral Spike proteins to the angiotensin-converting enzyme 2 that is largely expressed in vascular endothelium cells. The virus passage from the bloodstream to the epithelial tissue produces an inflammatory process, increasing the diapedesis of neutrophils and lymphocytes to the underlying connective tissue.^44,45^

González et al. proposed two distinct pathophysiological mechanisms to explain the appearance of different COVID-19 skin manifestations. The exanthema multiforme- and varicella-like manifestations have been associated with direct cytopathic effect of the virus on skin cells, leading to vacuolar degeneration and inducing keratinocyte apoptosis. Otherwise, Chilblain-like lesions have been associated with events similar to macrophage activation syndrome, such as plasminogen activator release by macrophages, and other findings as antiphospholipid antibodies.^48^

Chilblain-, erythema multiforme-, and varicella-like lesions appeared in a mean time of 7.6, 9.5, and 12 days after viral infection, respectively. Considering that SARS-CoV-2 incubation period varies from 1 to 14 days, the presence of these cutaneous manifestations should raise suspicion for the possibility of patient's contamination.^49,50^

Cutaneous manifestations mean durations were 17.5 days for Chilblain-like lesions, 10.3 days for erythema multiforme-like lesions, and 7 days for varicella-like lesions. The appearance and duration time of the lesions did not predict the severity of the infection.^45–51^

Histopathological analysis of the lesions was not performed in most studies. In cases of Chilblain like or erythema multiforme like with a histopathological examination, perivascular and superficial perieccrine lymphocytic infiltrate, lymphocytic vasculitis, and, sometimes, epidermal necrosis with erythrocyte extravasation were observed.^52,53^ There was no consensus regarding the indication of biopsies in COVID-19 pediatrics clinical practice. Many publications were from non-dermatologists, and the histopathological descriptions when available were not as detailed. Dermatologists might postulate that Chilblain-like versus erythema multiforme-like lesions would have different histopathological features, pathogenesis, clinical settings, and perhaps even prognostic implications. There was no report in the literature regarding histopathological analysis of varicella-like lesions.

The authors emphasize the importance of pediatric careful evaluation on cutaneous manifestations as a possible first sign of COVID-19 disease. Pediatric population might present a hyperinflammatory response due SARS-CoV-2 infection and early diagnosis may prevent possible complications such as Kawasaki disease, immune/idiopathic thrombocytopenic purpura, hemophagocytic lymphohistiocytosis, and multisystemic inflammatory syndrome.^53–55^

Among the limitations of this review, all data presented were obtained from articles published in literature; therefore, they do not represent all pediatric cases of COVID-19 with cutaneous manifestations. In addition, the authors point out the possibility that only the most relevant cases might have been reported in the available publications, which underestimates mild skin manifestations. Another limitation found during the review was that not all selected articles complained with all the information under study. Thus, these three items may have behaved as confounding factors affecting the findings of this review.

COVID-19 has no specific dermatological manifestation. The Chilblain-like lesions were the most common in pediatric patients. As in Chilblain-like lesions, the erythema multiforme-like lesions were described in this population as an acute, self-limited, manifestation. Varicella-like lesions were rarely reported in the literature; however, their presence should raise suspicion of SARS-CoV-2 infection in the current scenario.

Cutaneous symptoms related to COVID-19 remain relatively unknown by many health care professionals due to a shortage of literature reviews, especially regarding children and adolescents. This review consolidates available data and summarizes information of the COVID-19 pediatric skin manifestations, contributing to the development of new guidelines by pediatric societies. An increase in awareness and identification of these lesions may be essential to an earlier diagnosis and, consequently, the possibility of promptly care adoption as well as to interrupt the new coronavirus transmission chains in the current pandemic context. Further studies are important to better characterize their prevalence and relation to disease severity.
